# Spatiotemporal dynamics of crop water use and groundwater depletion under the winter wheat–summer maize rotation system in Henan Province, China

**DOI:** 10.3389/fpls.2025.1728535

**Published:** 2025-12-11

**Authors:** Zhao Zhang, Jing Wang, Jing Ning, Jinsong Ti

**Affiliations:** College of Tobacco Science, Henan Agricultural University, Zhengzhou, China

**Keywords:** double-cropping system, groundwater depletion, irrigation water requirement, sustainable agriculture, water balance framework

## Abstract

**Introduction:**

Henan Province plays a pivotal role in ensuring China’s food security through its extensive winter wheat–summer maize rotation system. However, excessive groundwater extraction has become a critical constraint on sustainable agricultural development.

**Methods:**

This study quantitatively analyzed the spatiotemporal dynamics of crop water use and groundwater depletion across Henan from 1961 to 2020 using a water balance framework that integrates crop evapotranspiration (ETc), effective precipitation (Re), irrigation water requirement (Iwr), and net groundwater consumption (NGWC).

**Results and Discussion:**

Results showed distinct and opposite trends in ETc between the two crops: winter wheat exhibited increasing ETc under climate warming, while summer maize experienced a decline driven by improved water-use efficiency. Effective precipitation decreased during key growth stages, leading to increased irrigation demand for winter wheat but a reduction for summer maize. Winter wheat remained the dominant driver of groundwater depletion, consuming three to four times more groundwater annually than maize. However, since 2010, maize groundwater use has risen sharply, intensifying water stress in the central–northern plains. To alleviate overexploitation, a dual strategy combining winter wheat fallowing and summer maize water conservation is proposed. These findings provide a scientific foundation for optimizing cropping patterns and improving water resource sustainability in major grain-producing regions of North China.

## 1 Introduction

Henan Province, located in the core of the North China Plain, is one of China’s most important grain production regions, supporting national food security through its extensive winter wheat–summer maize rotation system (Liu et al., 2013). The high multiple-cropping index and intensive use of fertilizer and irrigation have substantially increased land productivity, but they also rely heavily on groundwater abstraction to buffer the pronounced mismatch between crop water demand and uneven precipitation in this semi-humid to semi-arid environment (Wang and Shi, 2024b). Over the past several decades, continuous overdraft has led to widespread groundwater level decline, land subsidence, river flow reduction and degradation of associated ecosystems, making groundwater depletion a key constraint on the long-term sustainability of agricultural production in Henan and across the broader North China Plain (Du et al., 2024; Famiglietti, 2014).

In response to these challenges, considerable research in the field of agricultural water management has focused on quantifying crop water use, irrigation demand and groundwater stress in intensively cultivated regions. At the field and regional scales, water balance models, remote-sensing–driven approaches and water footprint assessments have been used to estimate reference evapotranspiration, crop evapotranspiration (*ET_c_*), effective precipitation (*R_e_*), and irrigation water requirement (*I_wr_*) for major cereal crops, and to evaluate their implications for blue-water consumption and water scarcity (D’odorico et al., 2020; Li et al., 2024b; Wang et al., 2023; Li et al., 2023). In the North China Plain, numerous studies have reported increasing evaporative demand for winter wheat under climate warming, contrasted with declining or stabilized *ET_c_* for summer maize due to the combined effects of the evaporation paradox and improved varieties and management (Ti et al., 2018). Parallel work using hydrological and groundwater-flow models has revealed large-scale depletion of aquifers driven by irrigation, and has highlighted the need for more efficient cropping patterns and irrigation strategies in major grain-producing regions (Cao et al., 2013; Gleeson et al., 2020; Tan et al., 2023).

Despite this progress, several important knowledge gaps remain for Henan’s winter wheat–summer maize rotation system. First, most existing studies have treated winter wheat and summer maize separately, or have only analyzed a single crop or limited time periods, making it difficult to capture the coupled and potentially compensating effects of the two crops on groundwater over multi-decadal scales (Mo et al., 2017; Liu et al., 2017). Second, while *ET_c_*, *R_e_* or *I_wr_* have been widely examined, relatively few studies have integrated these components into a unified water balance framework that explicitly links crop water consumption to net groundwater consumption (*NGWC*) and its spatial redistribution within double-cropping systems (Zhao et al., 2021). Third, the seasonal shift of groundwater stress within the rotation—particularly the evolving contribution of summer maize in the context of policy-driven yield improvement and climate variability—has not been systematically quantified using long time series and stage-resolved indicators. Finally, although winter wheat fallowing has been proposed as a promising measure to alleviate groundwater depletion in high-risk zones, there is still a lack of empirical evidence that simultaneously evaluates the hydrological consequences of winter wheat fallowing and summer maize water conservation within a consistent analytical framework (Cao et al., 2020; Kang et al., 2017; Pereira et al., 2002).

Addressing these gaps is crucial for designing rotation-specific water-saving strategies that reconcile groundwater protection with food production targets. By jointly considering both crops over several decades, and by explicitly quantifying irrigation water demand and *NGWC* at different growth stages and spatial scales, it is possible to reveal whether groundwater stress is merely concentrated in winter wheat, or is progressively transferring and amplifying within the summer maize season under changing climatic and management conditions. Such insights are essential for refining fallow policies, optimizing crop structure and improving irrigation scheduling in Henan Province and similar double-cropping regions worldwide.

Therefore, this study focuses on the winter wheat–summer maize rotation system in Henan Province from 1961 to 2020 with three specific objectives: (1) to quantify the long-term spatiotemporal patterns of *ET_c_*, *R_e_* or *I_wr_* for winter wheat and summer maize under the rotation system; (2) to estimate stage-based and decadal *NGWC* and annual net groundwater consumption (*ANGWC*) for both crops and to reveal how groundwater depletion has evolved across space and time; and (3) to assess the implications of alternative cropping and irrigation strategies, including winter wheat fallowing and summer maize water conservation, for relieving groundwater stress and supporting sustainable agricultural water management in this major grain-producing region.

## 2 Materials and methods

### 2.1 Study area and regional characteristics

**Figure 1:**
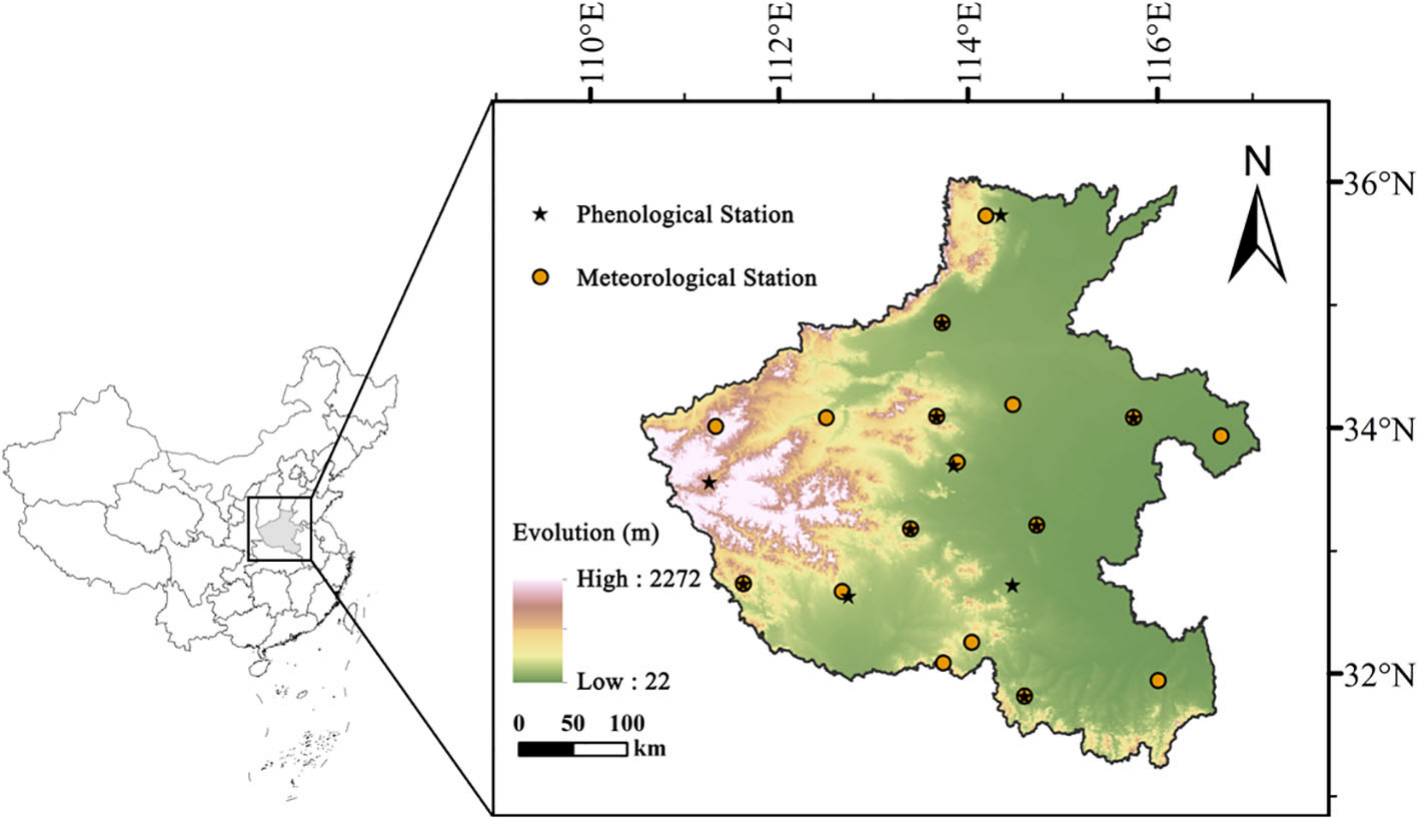
Geographical distribution of meteorological stations and altitude in Henan Province, China.

Henan Province, located in central-eastern China on the North China Plain, is one of the nation’s most important agricultural regions, with extensive wheat–maize double-cropping systems forming the backbone of local food production. The province spans 18 prefecture-level cities and covers diverse climatic and soil conditions, ranging from warm temperate semi-humid zones in the north to subtropical monsoon regions in the south (**Figure 1**).

Annual precipitation generally increases from northwest to southeast, while temperature and evaporation display inverse gradients. Soils are dominated by alluvial, brown, and yellow-brown types, providing a favorable environment for both summer maize and winter wheat cultivation. This climatic and edaphic heterogeneity underpins Henan’s role as a major grain production base, while also shaping regional differences in irrigation demand and groundwater dependence.

### 2.2 Data sources

This study utilized a 60-year dataset (1961–2020) integrating meteorological, agricultural, and crop-specific data. Meteorological data were obtained from 17 stations of the China Meteorological Network (http://data.cma.cn), encompassing variables such as daily temperature, wind speed, sunshine duration, precipitation, and humidity. Crop data, including growth-period information and crop coefficients, were derived from the China Meteorological Yearbook and the Food and Agriculture Organization of the United Nations (*FAO*) (China Meteorological Yearbook Editorial Department, 1961-2020; Allen et al., 1998). County-level sown area data were extracted from municipal statistical yearbooks to estimate spatial distributions of crop cultivation (China Agricultural Yearbook Editorial Committee, 1961-2020).

### 2.3 Calculation of crop water requirements

Reference crop evapotranspiration (*ET_0_*) was calculated using the *FAO*-recommended Penman–Monteith model (Allen et al., 1998) (Equation 1):

(1) $$ET_{0}=\frac{0.408\text{Δ}(R_{n}-G)+\frac{900}{T+273}u_{2}(e_{s}-e_{a})}{\text{Δ}+\gamma (1+0.34u_{2})}$$

where *ET_c_* is reference evapotranspiration (mm·day^-1^); *R_e_* is net radiation (MJ·m^-2^·day^-1^), calculated based on solar radiation derived from sunshine duration data; *G* is soil heat flux, considered negligible (G ≈ 0) for daily calculation steps; *T* is air temperature (°C); *u_2_* is wind speed at 2 m (m·s^-1^); e_s_ is the saturation vapor pressure (kPa), calculated from air temperature; e_a_ is the actual vapor pressure (kPa), derived from relative humidity data; *Δ* is the slope of the vapor-pressure curve (kPa·°C^-1^); and *γ* is the psychrometric constant (kPa·°C^-1^).

Crop evapotranspiration (*ET_c_*) was derived as Equation 2:

(2) $$ET_{c}=ET_{0}\cdot K_{c}$$

where *K_c_* is the crop coefficient.

Effective precipitation (*R_e_*) represents the portion of rainfall retained within the root zone sufficient for crop use (Kang and Cai, 1996) (Equation 3):

(3) $$R_{e}=\alpha_{i}\cdot P_{i}$$

where *P_i_* is total precipitation (mm) and *α_i_* is the effective utilization coefficient:

(4) $$\{\begin{array}{l} P_{i}\leq 5mm,\;\;\;\;\;\;\;\;\;\;\;\;\;\;\;\;\;\;\;\;\alpha_{i}=0\;\;\;\;\;\;\\ 5mm\leq P_{i}\leq 50mm,\;\;\;\;\;\;\;\;\;\;\alpha_{i}=0.90\\ P_{i}>50mm,\;\;\;\;\;\;\;\;\;\;\;\;\;\;\;\;\;\;\;\alpha_{i}=0.75 \end{array}$$

The fixed coefficient method employed for estimating effective precipitation (*R_e_*), while operationally straightforward and widely applied in large-scale, long-term hydrological assessments, possesses inherent limitations, as shown in Equation 4. This simplified approach does not explicitly account for the dynamic influences of soil hydraulic properties (e.g., texture, structure, water-holding capacity), topography, or rainfall intensity and duration on the actual partitioning of rainfall into infiltration, runoff, and deep percolation. Consequently, this method may introduce systematic biases in *R_e_* estimation across different pedo-climatic zones. For instance, in regions dominated by sandy soils with high infiltration rates and low water-holding capacity, the fixed coefficients might overestimate the fraction of precipitation retained within the root zone available for crop use. Conversely, in areas with clayey soils characterized by higher water retention but potentially slower initial infiltration, the method could underestimate *R_e_*, particularly for low-intensity rainfall events. These potential inaccuracies in *R_e_* would propagate directly into the subsequent calculations of irrigation water requirement (*I_wr_*) and *NGWC*, likely leading to an overestimation of *I_wr_* and *NGWC* in sandy soil areas and an underestimation in clayey soil regions. Despite these limitations, the method provides a consistent and computationally efficient framework for analyzing long-term spatiotemporal trends across the extensive and diverse study area. The findings and conclusions of this study should therefore be interpreted with an understanding of this methodological simplification, which highlights a valuable direction for future research to incorporate more process-based soil water balance models.

Irrigation water requirement (*I_wr_*) was then estimated as Equation 5:

(5) $$I_{wr}=ET_{c}-R_{e}$$

Positive *I_wr_* indicates irrigation demand exceeding rainfall; negative values imply rainfall sufficiency.

### 2.4 Inverse distance weighted interpolation

Spatial interpolation of meteorological-station data to surface scale was achieved using the inverse distance weighting method (Rolim et al., 2011):

(6) $$Z=\frac{\sum_{i=1}^{n}(\frac{1}{d_{i}^{k}})Z_{i}}{\sum_{i=1}^{n}(\frac{1}{d_{i}^{k}})}$$

where *Z* is the interpolated value, *Z_i_* is the observed value at station *i*, *d_i_* is the distance to the interpolation point, *n* is the number of stations (typically 3), and *k* = 2 is the distance-decay parameter (Equation 6).

### 2.5 Estimation of net groundwater consumption

Net groundwater consumption quantifies the average groundwater volume consumed per unit area by crops over their growth cycle, as shown in Equation 7:

(7) $$NGWC=\frac{\sum_{j=1}^{m}\sum_{i=1}^{n}(R_{e,i,j}-ET_{c,i,j})}{m\cdot n}$$

where *R_e,i,j_* is effective precipitation, *ET_c,i,j_* is crop water requirement, *m* is the number of years, and *n* is the number of growth stages. Positive values denote groundwater recharge; negative values indicate net depletion.

Annual net groundwater consumption (*ANGWC*) represents the total volume of groundwater consumed by a specific crop within a given region over a defined time period. To obtain stable and representative results at the county scale, and to minimize the potential influence of extreme climatic events (such as severe droughts or floods) on crop water use in a single year, a decadal mean approach was adopted for the calculation of *ANGWC*.

In this method, the decadal average *NGWC* of summer maize and winter wheat was first calculated for each ten-year period (e.g., 1981–1990, 1991–2000, 2001–2010, and 2011–2020). The decadal mean *NGWC* was then multiplied by the sown area of the corresponding mid-point year of each decade (e.g., 1985, 1995, 2005, and 2015) to estimate the representative *ANGWC* at the county level. This approach can be expressed as Equation 8:

(8) $$ANGWC_{c,k}={\bar{NGWC}}_{c,k}^{10yr}*A_{c,k}^{mid}$$

where $ANGWC_{c,k}$ denotes the annual net groundwater consumption of crop c (winter wheat or summer maize) in county k; $\bar{NGWC}$ represents the ten-year average net groundwater consumption (mm) for crop c; and $A_{c,k}^{mid}$ is the cultivated area (ha) of the mid-point year of the corresponding decade.

By linking decadal mean groundwater consumption intensity with the representative cropping structure of each period, this approach effectively reduces the interference of short-term climate variability and interannual fluctuations. It provides a more robust and realistic representation of the long-term dynamics of groundwater consumption in Henan’s wheat–maize rotation system, ensuring that the derived county-level ANGWC values reflect both hydrological and agronomic conditions in a balanced and scientifically consistent manner.

### 2.6 Mann–Kendall Trend test and Sen’s slope estimation

#### 2.6.1 Mann–Kendall trend test

The Mann–Kendall (*M-K*) test is a non-parametric method for detecting monotonic trends in time-series data (Kendall, 1957) (Equation 9):

(9) $$S=\sum_{i=1}^{n-1}\sum_{j=i+1}^{n}\mathrm{sgn}(x_{j}-x_{i})$$

With Equation 10

(10) $$\mathrm{sgn}(x_{j}-x_{i})=\{\begin{array}{c} +1,if\;x_{j}-x_{j}>0\\ 0,if\;x_{j}-x_{j}=0\\ -1,if\;x_{j}-x_{j}<0 \end{array}$$

The variance is given by Equation 11:

(11) $$Var(S)=\frac{n(n-1)(2n+5)-\sum_{i=1}^{m}t_{i}(t_{i}-1)(2t_{i}+5)}{18}$$

and the standardized statistic Z_s_ is computed as Equation 12:

(12) $$Z_{S}=\{\begin{array}{c} \frac{S-1}{\sqrt{Var(S)}},if\;S>0\\ 0,if\;S=0\\ \frac{S-1}{\sqrt{Var(S)}},if\;S<0 \end{array}$$

At a 5% significance level (*α* = 0.05), trends are significant when *|Z_s_|* > 1.96.

#### 2.6.2 Sen’s slope estimation

Sen’s method estimates the magnitude of a trend by computing the median of all pairwise slopes (Sen, 1968) (Equation 13):

(13) $$b=Median(\frac{x_{j}-x_{z}}{j-1})$$

where *x_j_* and *x_z_* are data at time points *j* and *z* (*j* > *z*), and *b* represents the annual change rate. The approach is robust to outliers and widely used to quantify changes in climatic and hydrological variables.

### 2.7 Statistical analysis

All meteorological and agricultural data were processed using RStudio and Microsoft Excel 2021. Spatial analyses and visualizations were performed in ArcGIS, Origin 2021, and Excel 2019, enabling clear representation of the spatiotemporal evolution of evapotranspiration, irrigation demand, and groundwater consumption for both crops.

## 3 Results

### 3.1 Spatiotemporal distribution of crop evapotranspiration

**Figure 2:**
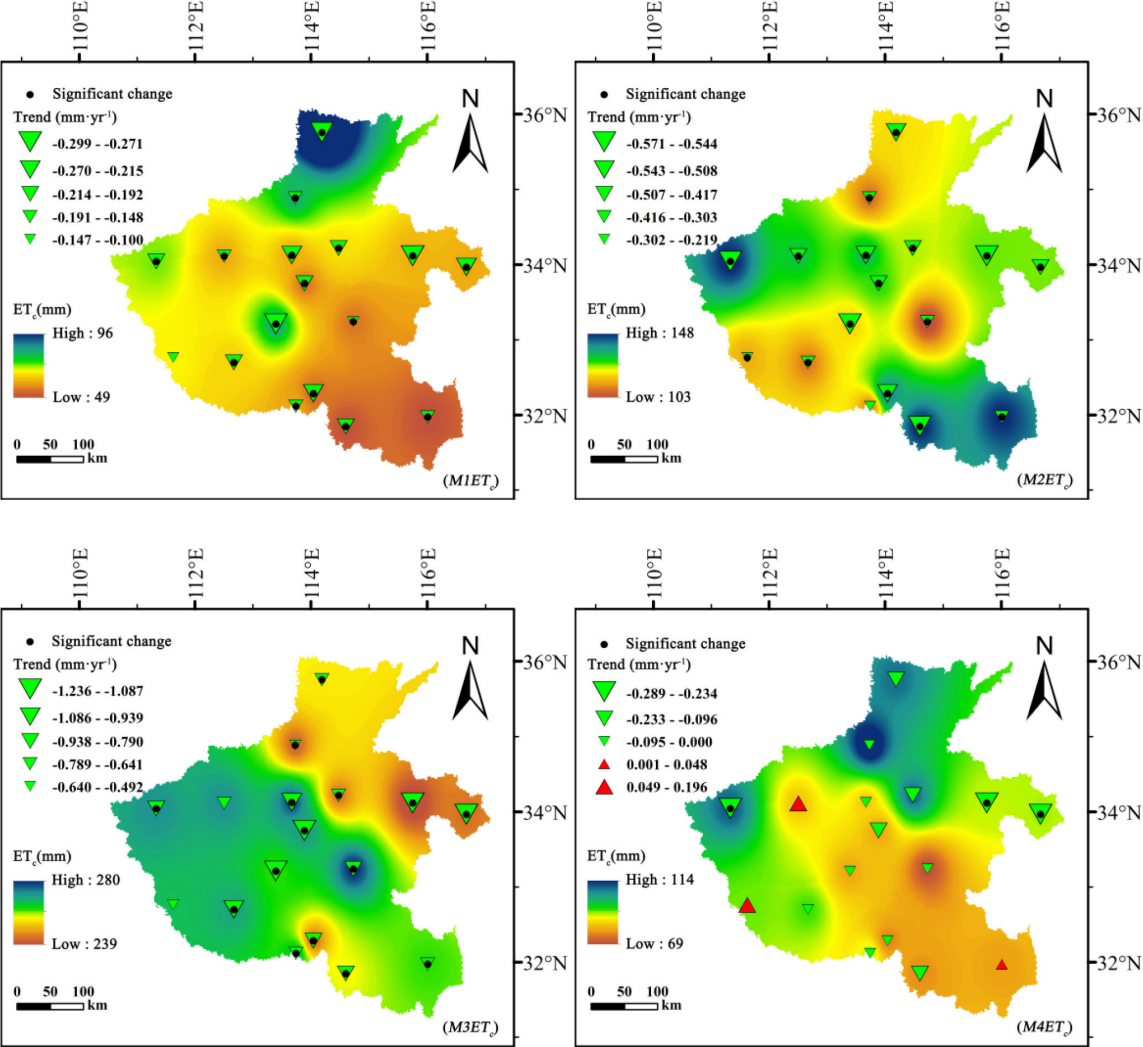
Spatiotemporal variation of *ET_c_* (mm) for summer maize across different growth stages in Henan Province, 1961–2020.

**Figure 3:**
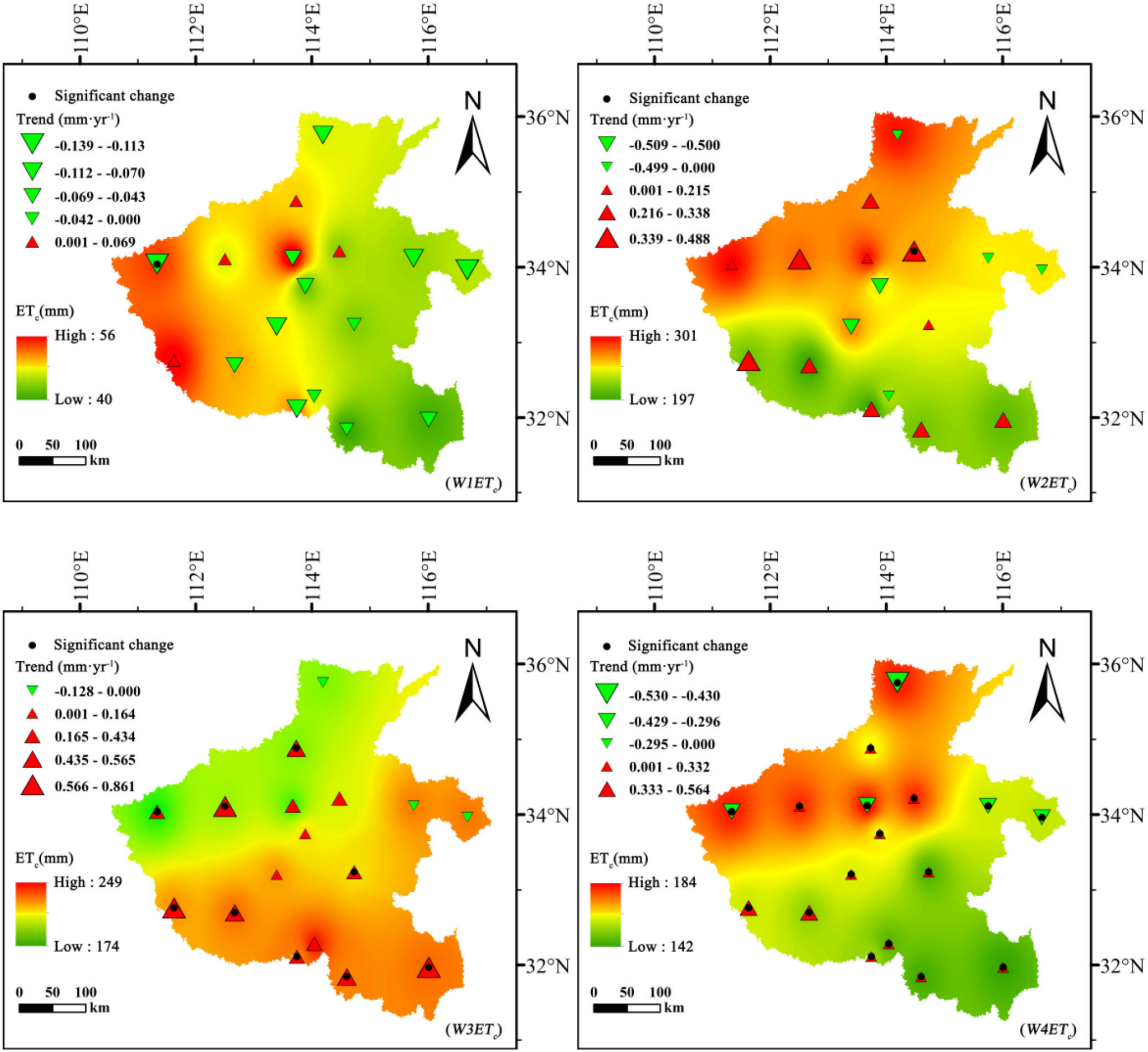
Spatiotemporal variation of *ET_c_* (mm) for winter wheat across different growth stages in Henan Province, 1961–2020.

During 1961–2020, crop evapotranspiration (*ET_c_*) for summer maize and winter wheat in Henan Province exhibited pronounced spatiotemporal differentiation and strikingly opposite temporal trends (**Figures 2**, **3**). Summer maize showed a consistent downward trend in *ET_c_* across all growth stages, with the most substantial decline occurring during the mid-growth stage (*M3ET_c_*), where Sen’s slope reached −1.236 mm·year^-1^. Significant decreases were also observed during the development (*M2ET_c_*) and early growth (*M1ET_c_*) stages, suggesting a gradual reduction in atmospheric evaporative demand or improved water-use efficiency.

In contrast, winter wheat exhibited a distinct increase in *ET_c_*, particularly during the development (*W2ET_c_*) and mid-growth (*W3ET_c_*) stages. In some central and northern regions, this increase was statistically significant (|Z| > 1.96), indicating regionally robust upward trends. This opposite pattern of *ET_c_* evolution between the two crops highlights their differing responses to climate change and irrigation practices.

Spatially, both crops displayed peak water requirements during their mid-growth stages, but the spatial patterns and magnitudes differed markedly. Summer maize *ET_c_* reached a maximum of 280 mm, with high-value zones concentrated in the central and northern plains, including Zhengzhou, Xuchang, and Luohe, areas characterized by relatively high solar radiation and strong evapotranspiration potential. Winter wheat reached its peak *ET_c_* during the development stage (max: 301 mm), with high values distributed across the central, western and northern plains, where temperature and radiation levels rise sharply during spring. The broader range of *ET_c_* variation in winter wheat indicates its greater sensitivity to local meteorological fluctuations.

Notably, the increasing *ET_c_* trend observed for winter wheat coincided with a declining trend in effective precipitation, indicating a growing mismatch between water demand and natural supply. This imbalance has intensified irrigation dependence during the critical stages of tillering and heading. Conversely, the declining *ET_c_* in summer maize, combined with a moderate rise in early-season precipitation, reflects a relatively improved water supply–demand relationship. These opposite patterns underscore a seasonal shift in water stress within the rotation system.

### 3.2 Spatiotemporal distribution of effective precipitation

**Figure 4:**
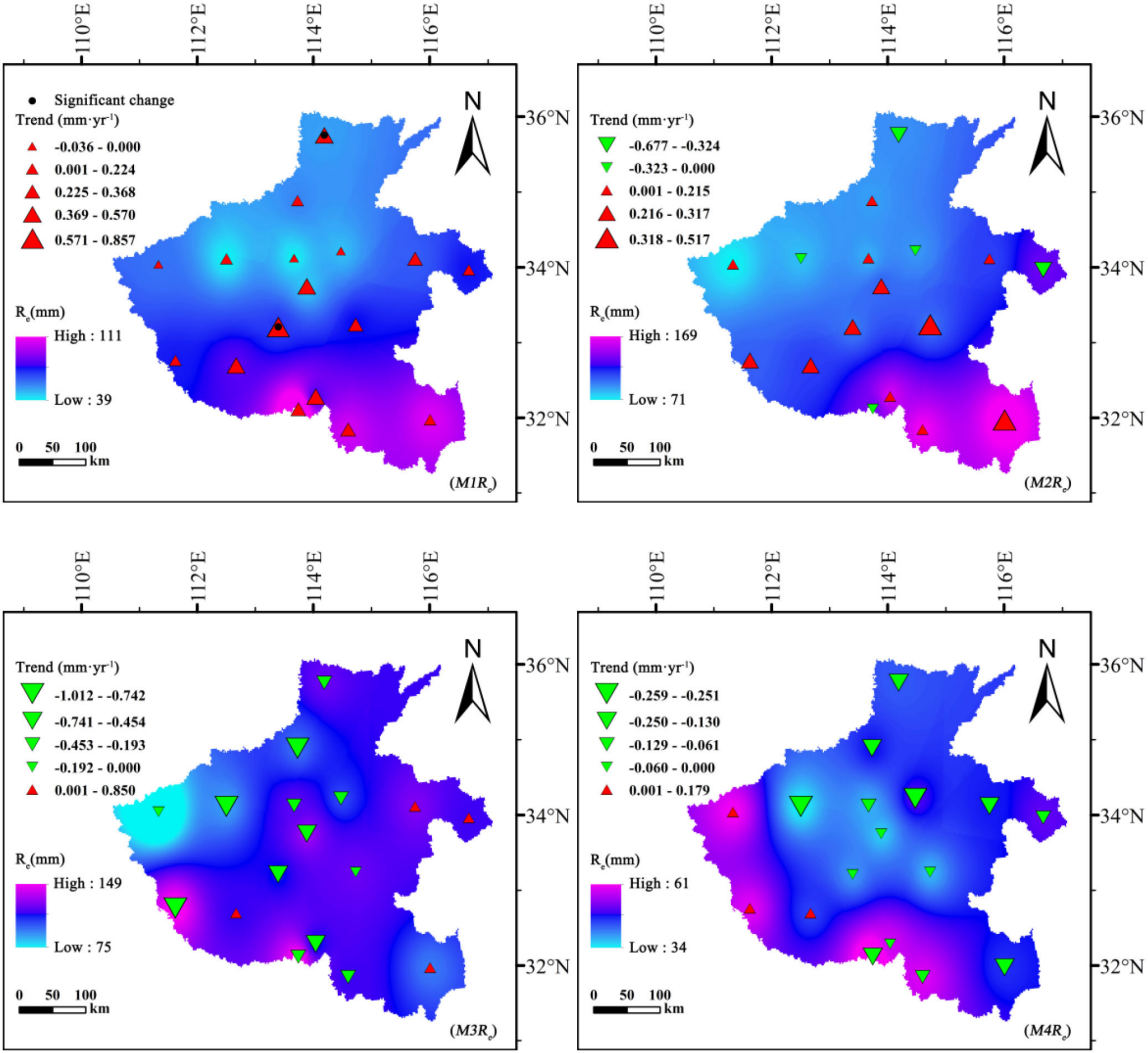
Spatiotemporal variation of effective precipitation (*R_e_*, mm) for summer maize across different growth stages in Henan Province, 1961–2020.

**Figure 5:**
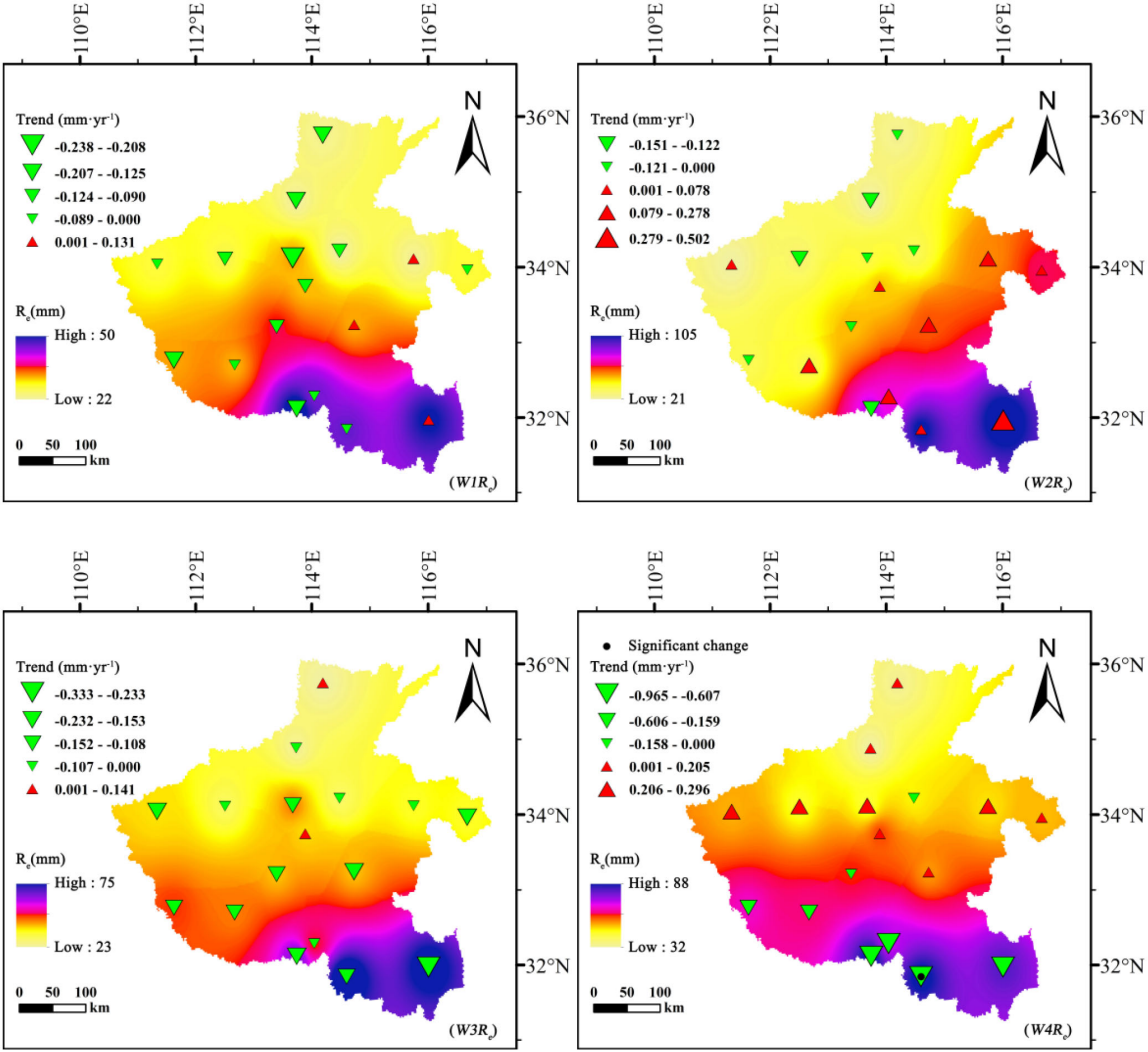
Spatiotemporal variation of effective precipitation (*R_e_*, mm) for winter wheat across different growth stages in Henan Province, 1961–2020.

Effective precipitation (*R_e_*) during the growth stages of summer maize and winter wheat exhibited complex spatiotemporal dynamics across Henan Province (**Figures 4**, **5**). For summer maize, *R_e_* displayed distinct phased characteristics. During the early growth stage (*M1R_e_*), a significant upward trend was observed across most of the province, suggesting an improvement in moisture conditions during the seedling establishment phase. However, as crop development progressed, spatial divergence in *R_e_* became increasingly evident. By the mid-growth (*M3R_e_*) and maturity (*M4R_e_*) stages, a clear and consistent downward trend dominated the spatial pattern, with the most significant reduction during the mid-growth stage (Sen’s slope = −1.012 mm·year^-1^). This indicates that rainfall during the main water consumption period of maize has gradually become less effective.

For winter wheat, *R_e_* variations showed a generally declining trend across all growth stages. The maturity stage (*W4R_e_*) exhibited the most pronounced and consistent decrease (Sen’s slope = −0.965 mm·year^-1^), followed by the mid-growth stage (*W3R_e_*). During the early (*W1R_e_*) and development (*W2R_e_*) stages, spatial differentiation was evident, with localized increases in southern areas but declines dominating the overall pattern.

Spatially, summer maize *R_e_* peaked during the development stage at 169 mm, followed by the mid-growth stage at 149 mm, exhibiting strong spatial heterogeneity throughout. High-value zones were mainly distributed in southern Henan. In contrast, winter wheat *R_e_* values were generally lower, with a maximum of 105 mm during the development stage and a minimum of 50 mm during the early stage. The relatively low and uneven precipitation during winter wheat’s growing period underscores its vulnerability to moisture deficits and explains its heavy reliance on groundwater irrigation.

Overall, these spatiotemporal variations in effective precipitation constitute a primary driver of the contrasting irrigation requirements and groundwater consumption patterns between summer maize and winter wheat.

### 3.3 Spatiotemporal distribution of irrigation water requirement

**Figure 6:**
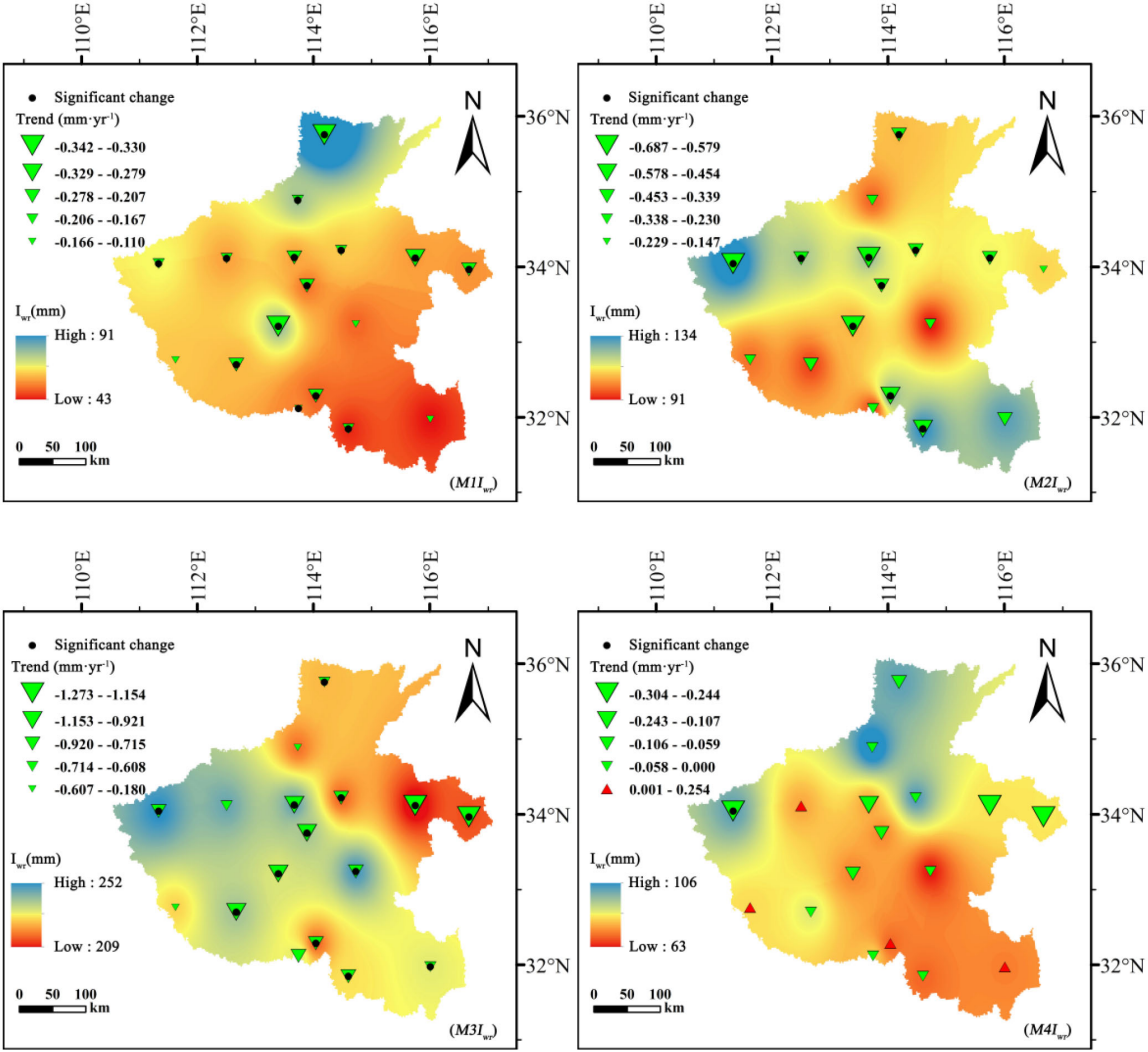
Spatiotemporal variation of irrigation water requirement (*I_wr_*, mm) for summer maize across different growth stages in Henan Province, 1961–2020.

**Figure 7:**
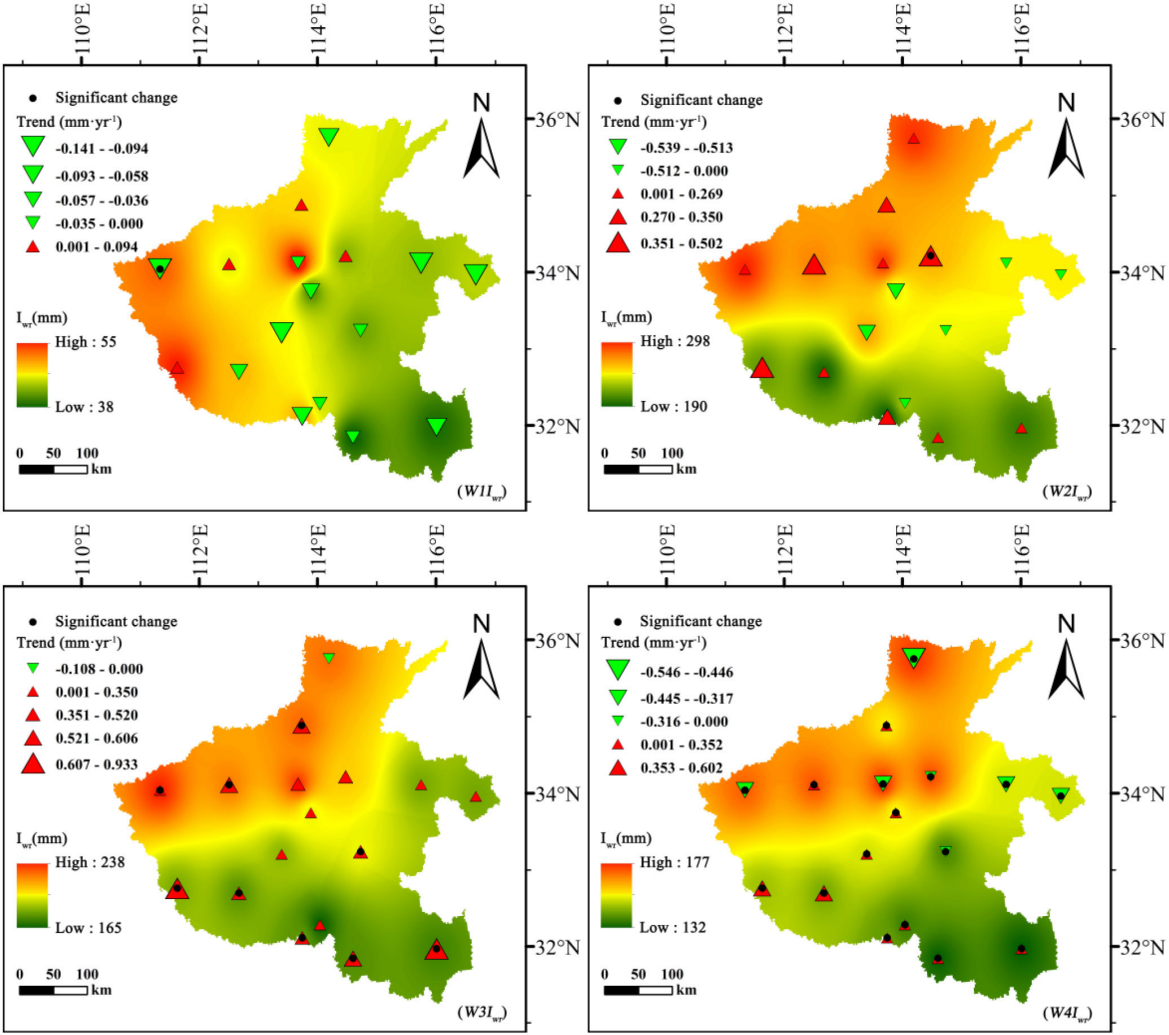
Spatiotemporal variation of irrigation water requirement (*I_wr_*, mm) for winter wheat across different growth stages in Henan Province, 1961–2020.

The irrigation water requirements (*I_wr_*) for summer maize and winter wheat exhibited markedly divergent trends between 1961 and 2020 (**Figures 6**, **7**). For summer maize, *I_wr_* displayed a persistent downward trend across all growth stages, consistent with the overall reduction in crop evapotranspiration and the moderate rise in effective precipitation during early growth stages. The most substantial decline occurred during the mid-growth stage (*M3I_wr_*), with a Sen’s slope of −1.273 mm·year^-1^. Similar downward tendencies were also evident during the early (*M1I_wr_*), development (*M2I_wr_*), and maturity (*M4I_wr_*) stages. Spatially, summer maize *I_wr_* was highly concentrated during the mid-growth stage, reaching a maximum of 252 mm, while remaining relatively low during the other stages. High-value zones were clustered mainly in the central and northern plains.

In contrast, winter wheat exhibited more complex and spatially heterogeneous patterns. During the early growth stage (*W1I_wr_*), most regions showed a declining trend, reflecting generally favorable moisture conditions during early winter. However, at the critical development stage (*W2I_wr_*), the pattern diverged sharply: while several regions, especially in central Henan, experienced a significant increase (|Z| > 1.96), others exhibited notable decreases. This spatial polarization reflects growing disparities in water supply–demand relationships across different subregions. During the mid-growth stage (*W3I_wr_*), the overall trend shifted towards increasing irrigation demand, implying a sustained intensification of water stress during this critical phase.

Spatially, winter wheat *I_wr_* peaked during the development stage, reaching 298 mm, and exhibited pronounced spatial heterogeneity throughout all stages. High-value zones were concentrated in the central and northern plains, which correspond closely to areas of severe groundwater overexploitation.

Overall, the contrasting trajectories of *I_wr_* between summer maize and winter wheat reveal a seasonal redistribution of irrigation pressure: maize irrigation demand is gradually easing, while wheat irrigation requirements continue to intensify.

### 3.4 Spatiotemporal distribution of net groundwater consumption

#### 3.4.1 Stage-based net groundwater consumption

**Figure 8:**
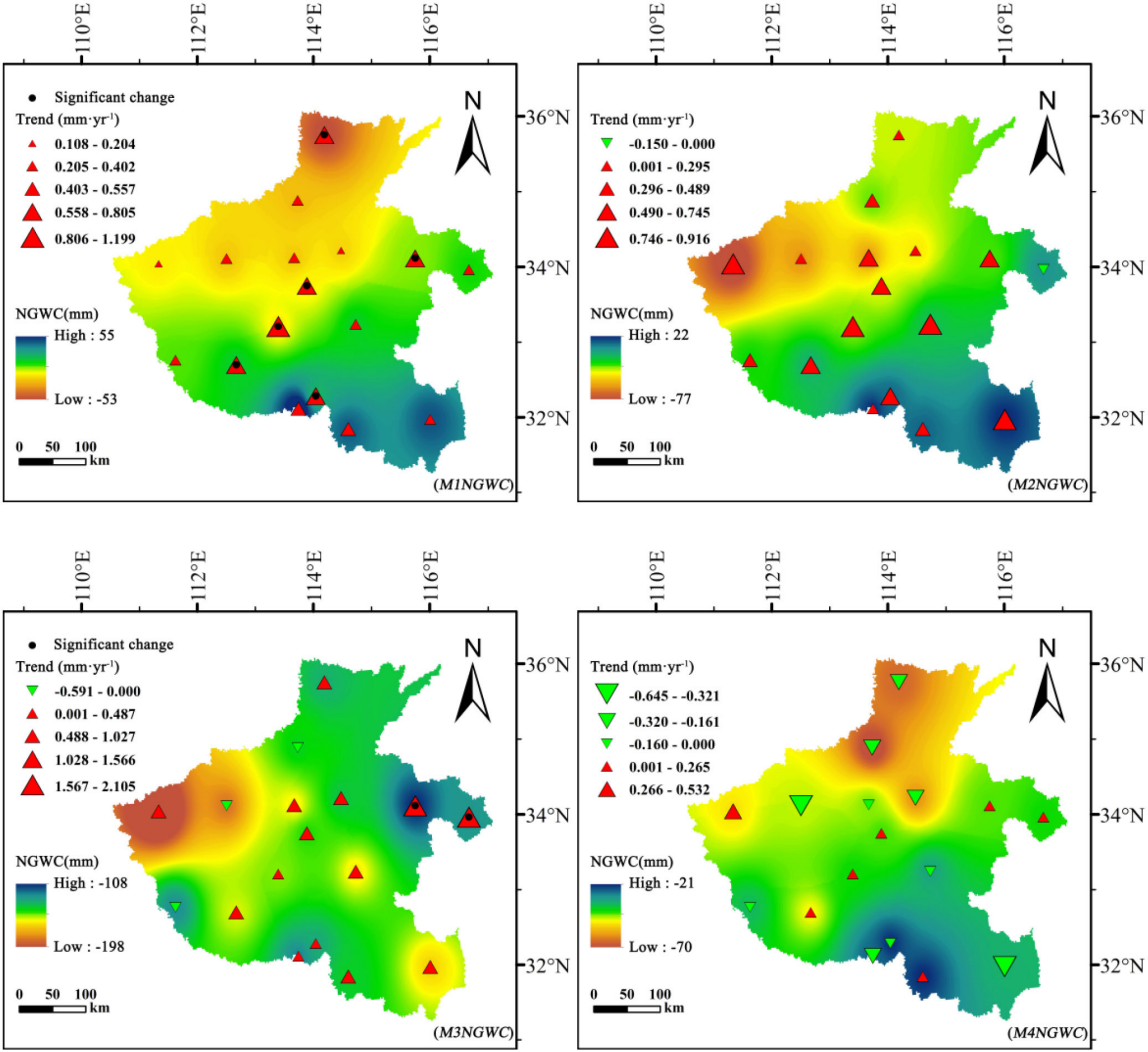
Spatiotemporal variation of net groundwater consumption (*NGWC*, mm) for summer maize across different growth stages in Henan Province, 1961–2020.

**Figure 9:**
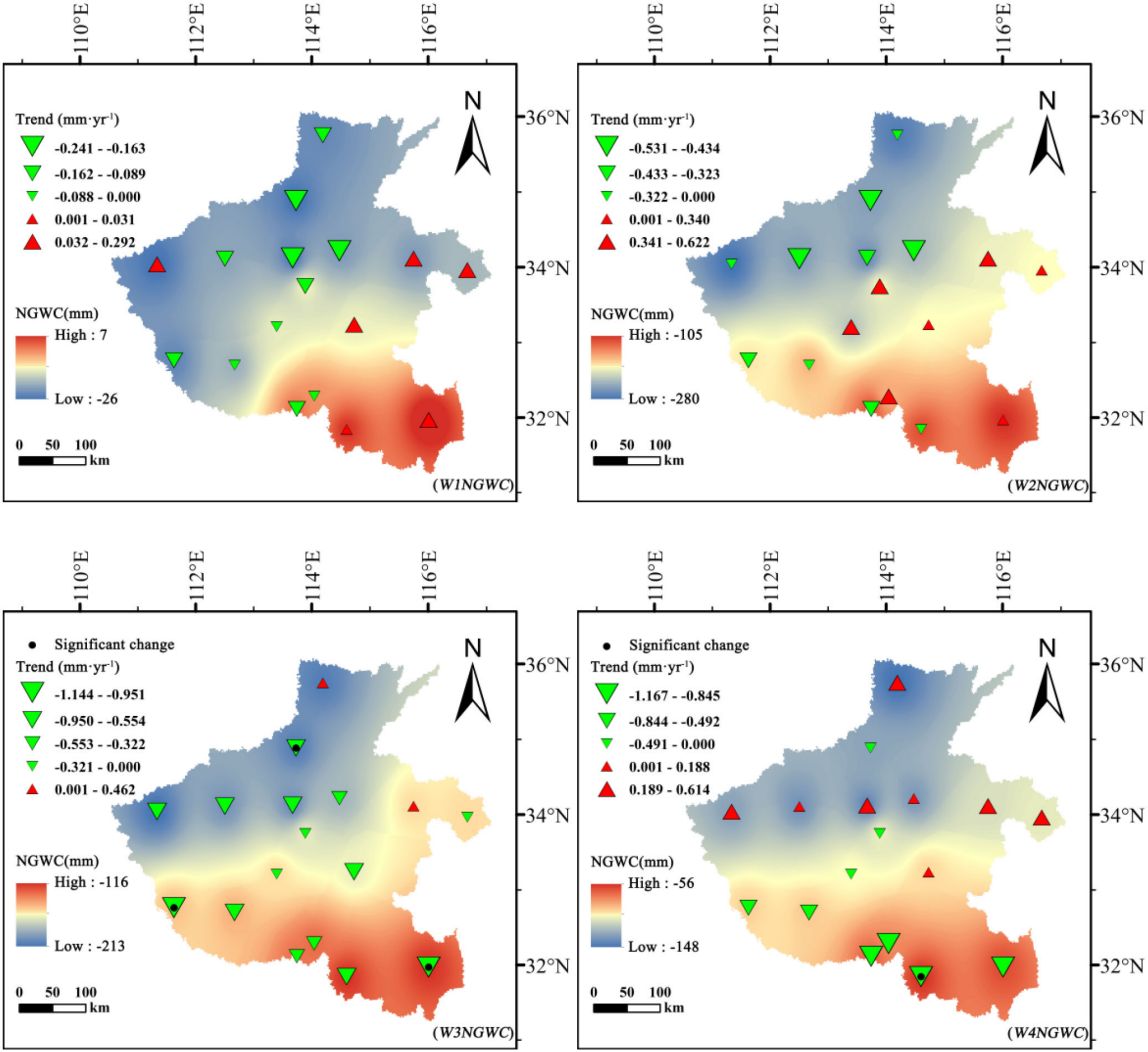
Spatiotemporal variation of net groundwater consumption (*NGWC*, mm) for winter wheat across different growth stages in Henan Province, 1961–2020.

Winter wheat and summer maize exhibited fundamentally different effects on groundwater dynamics and depletion trajectories (**Figures 8**, **9**). For winter wheat, *NGWC* values remained negative throughout all growth stages, indicating a persistent net consumption of groundwater across its entire life cycle. Interannual analyses revealed clear downward trends in *NGWC* during the mid-growth stage (*W3NGWC*) and maturity stage (*W4NGWC*), with Sen’s slopes reaching −1.144 mm·year^-1^ and −1.167 mm·year^-1^, respectively. These negative slopes reflect a progressive intensification of groundwater withdrawal during these critical phenological phases. The developmental stage (*W2NGWC*) displayed spatially mixed trends, with both positive and negative shifts, suggesting heterogeneous management practices and localized hydrological differences across regions.

In contrast, summer maize exhibited distinct phased behavior in *NGWC* evolution. During the initial stage (*M1NGWC*) and development stages (*M2NGWC*), most *NGWC* values were positive, indicating that effective precipitation not only satisfied crop water demands but also contributed net recharge to groundwater. However, by the mid-growth stage (*M3NGWC*), all *NGWC* values turned negative, identifying this phase as the critical period of net groundwater depletion for summer maize. Particularly noteworthy is the statistically significant upward trend in *NGWC* during the early growth stage (|Z| > 1.96), signifying a long-term transformation from “net recharge” to “net depletion”.

Spatially, winter wheat exhibited its greatest groundwater depletion during the development stage, reaching a minimum *NGWC* of −279.98 mm, with high-consumption zones concentrated in the central–northern plains. Similarly, summer maize showed high depletion levels in these same areas but displayed positive (recharge) values in the south, where precipitation and soil water retention are higher.

These results indicate that winter wheat is a net groundwater consumer throughout its entire growth period, whereas summer maize consumption is concentrated during the mid-growth stage. This difference in phased consumption patterns suggests that implementing precision irrigation management tailored to different crops and growth stages holds significant potential for optimizing water resource utilization.

#### 3.4.2 Decadal dynamics of annual net groundwater consumption

**Figure 10:**
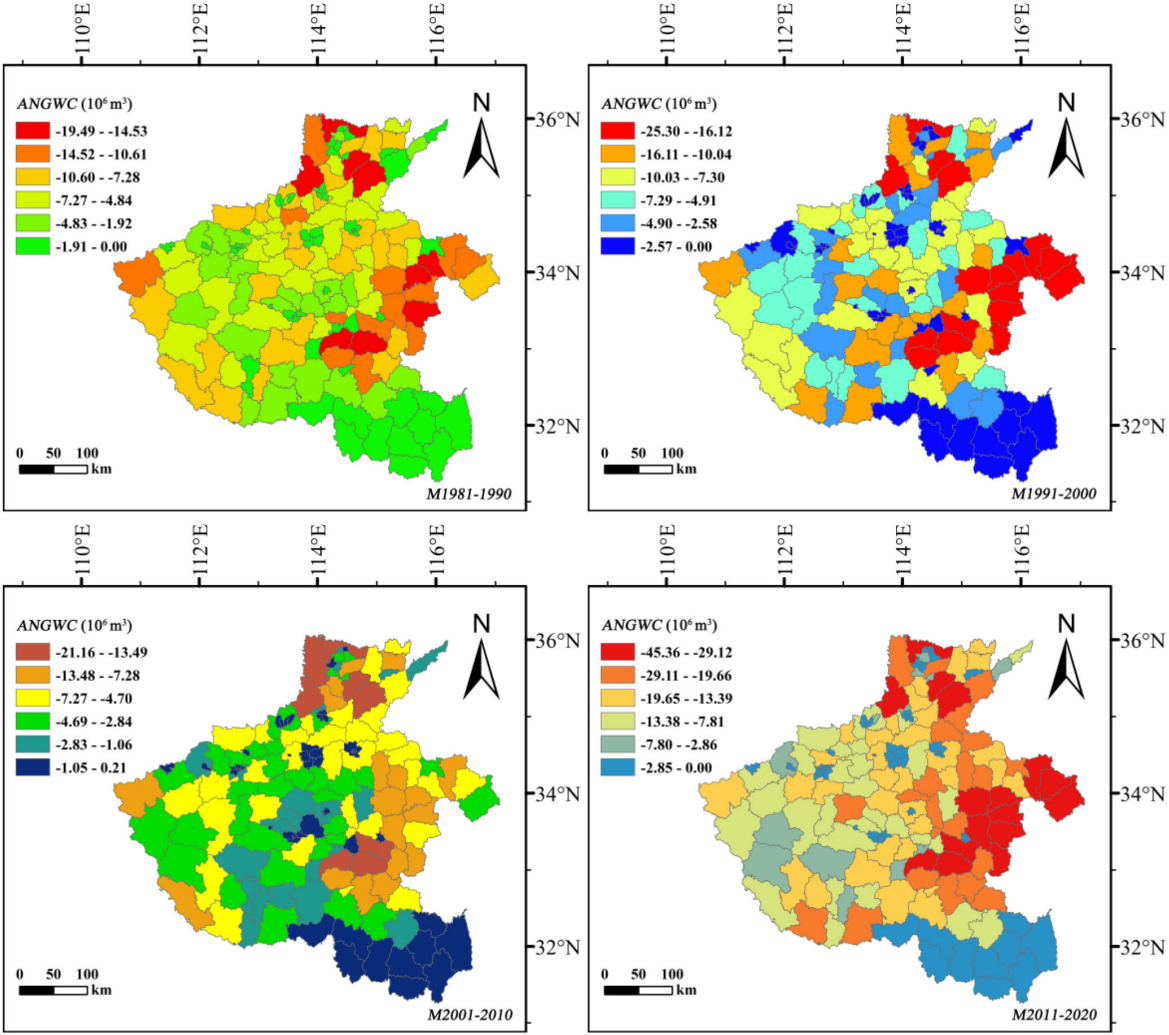
Spatiotemporal variation in annual net groundwater consumption (*ANGWC*, 10^6^ m^3^) of summer maize at the county scale in Henan Province, 1981–2020.

**Figure 11:**
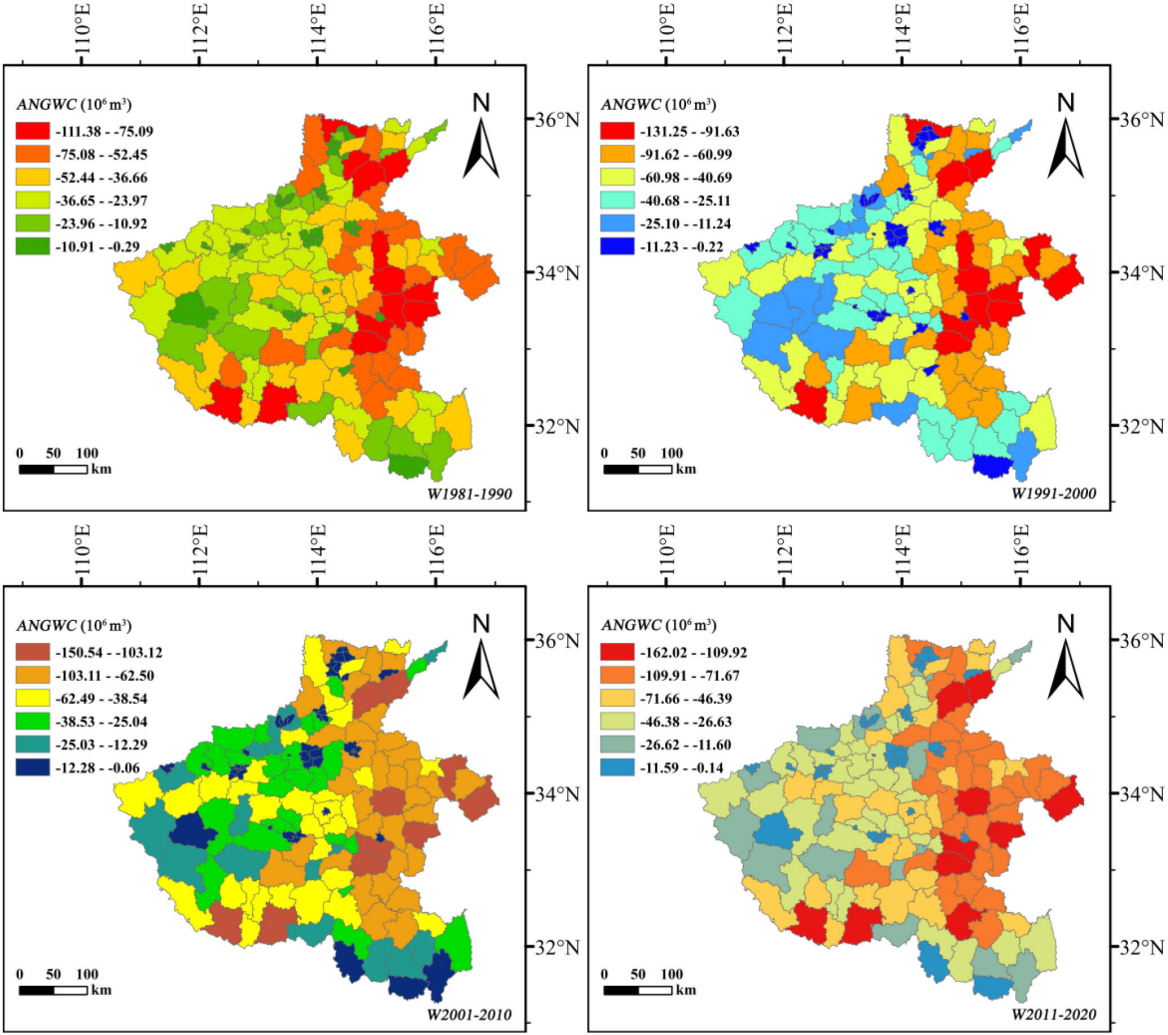
Spatiotemporal variation in annual net groundwater consumption (*ANGWC*, 10^6^ m^3^) of winter wheat at the county scale in Henan Province, 1981–2020.

To minimize the influence of extreme climatic years on groundwater assessment, decadal mean *NGWC* values were used to estimate county-level *ANGWC* (**Figures 10**, **11**). The results reveal contrasting decadal trajectories between the two crops. The *ANGWC* of summer maize remained relatively stable before 2010, fluctuating around –19.49 T (1 T = 10^6^ m^3^), but surged sharply to –45.36 T during 2011–2020—an increase exceeding 130%. This acceleration corresponds to both expanded cultivation and rising evapotranspiration deficits, indicating growing groundwater dependence in the post-2010 period. In contrast, winter wheat consistently dominated total groundwater depletion. Its *ANGWC* increased steadily from –131.25 T during 1991–2000 to –162.02 T in 2011–2020, remaining three to four times higher than that of summer maize.

Spatially, the central-northern plains emerged as persistent depletion hotspots throughout all decades, with overlapping high-consumption zones for both crops. These areas correspond to the region’s densest double-cropping belts and most intensive irrigation use, leading to long-term overextraction. The southern counties, characterized by higher precipitation and smaller irrigation reliance, displayed relatively balanced or even slightly positive *ANGWC*, indicating episodic groundwater recharge.

Overall, the decadal-scale evolution of *ANGWC* demonstrates that while winter wheat remains the primary driver of chronic groundwater depletion, the rapid post-2010 intensification in summer maize consumption has aggravated the total extraction pressure on aquifers. The superimposed effect of both crops in the central-northern region has transformed this zone into a critical area of groundwater stress.

## 4 Discussion

This study employs a water balance–based analytical framework to systematically elucidate the mechanisms underlying groundwater depletion within Henan Province’s winter wheat–summer maize rotation system over the past six decades. The results not only reaffirm the widespread agricultural water scarcity across the North China Plain but also reveal—through the opposite temporal trajectories of the two crops—a dynamic process of groundwater stress transfer and amplification within the rotation system itself (Piao et al., 2010). Furthermore, the post-2010 surge in groundwater depletion, particularly for summer maize, underscores the need for an integrated analysis that combines climatic, agronomic, and increasingly important policy and management drivers. These findings deepen the understanding of how climate variability and agronomic adaptation jointly shape groundwater consumption patterns in intensively cultivated double-cropping systems.

### 4.1 Drivers of changes in crop water requirements

This study demonstrates that the evapotranspiration (*ET_c_*) of winter wheat increased significantly during critical growth stages, whereas *ET_c_* of summer maize generally decreased. These divergent trajectories reflect the combined influences of climate change and agronomic adaptation (Zhang et al., 2013).

For winter wheat, the rising *ET_c_* is consistent with findings across the North China Plain, where increased air temperature and solar radiation during spring and early summer have elevated reference evapotranspiration (*ET_0_*) and thereby enhanced *ET_c_* (Mo et al., 2005). The spatial significance of this trend confirms that climate warming has systematically raised the evaporative potential of winter wheat fields (Tan et al., 2022).

Conversely, the decline in *ET_c_* for summer maize reflects both climatic moderation and technological improvement. The “evaporation paradox”, characterized by declining wind speed and reduced sunshine duration, has moderated *ET_0_* (Wang et al., 2007). Concurrently, advances in maize breeding have introduced drought-tolerant, short-duration, and high-efficiency cultivars with optimized canopy structures and improved transpiration efficiency (Chaves et al., 2003; Cao et al., 2015). These improvements, together with adaptive irrigation and soil management, have enhanced field-level water-use efficiency, offsetting the potential *ET_c_* increase from climatic warming (Zhang et al., 2017, 2024).

### 4.2 Drivers of effective precipitation variation

The evolution of effective precipitation (*R_e_*) in Henan Province reflects changing precipitation regimes and surface hydrological conditions. For summer maize, the early-growth-stage increase in *R_e_* is linked to interannual fluctuations in the East Asian summer monsoon (Ding et al., 2008). However, the subsequent decline during mid- and late growth stages indicates a structural change, where increasing rainfall concentration and extreme events promote runoff and percolation losses, reducing the fraction of rainfall effectively available to crops (Cao et al., 2013; Sun et al., 2022).

For winter wheat, the widespread decline in *R_e_* across all phenological stages corresponds to the long-term decrease in winter–spring precipitation and snowfall observed throughout North China. Spatial analysis further indicates that soil texture and structure exert a strong control over the spatial heterogeneity of *R_e_*. The brown and cinnamon soils dominating the central–northern plains of Henan, although possessing favorable tillage properties, have relatively low water-holding capacity compared with the yellow-brown and paddy soils prevalent in the southern region. Under persistently limited rainfall conditions, these coarse-textured soils exhibit a higher risk of desiccation and reduced infiltration efficiency, thereby amplifying the effects of meteorological drought and diminishing the effective utilization of precipitation (Kang et al., 2017).

This spatial coupling between soil hydro-physical properties and precipitation effectiveness explains why regions dominated by brown and cinnamon soils coincide with zones of severe water deficit in winter wheat production (Zhao et al., 2024). The results indicate that precipitation pattern shifts and soil-mediated infiltration limitations together govern the spatial distribution of water supply–demand imbalances across Henan’s wheat–maize rotation system (Wang et al., 2024a).

### 4.3 Drivers of irrigation water requirement variations

Variations in irrigation water requirement (*I_wr_*), the crucial link between surface processes and groundwater extraction, reflect the combined influences of climate forcing, agronomic adaptation, and water management capacity.

For summer maize, the consistent decline in *I_wr_* across all growth stages results from the joint effects of reduced *ET_c_* and increased early-season *R_e_*. This finding aligns with reports that water-saving technologies substantially mitigate maize irrigation requirements in the North China Plain (Kang et al., 2017).

Conversely, winter wheat exhibits increasing *I_wr_* during its critical growth phases, accompanied by pronounced spatial heterogeneity. This upward trend arises from the widening gap between water demand and supply—driven by higher *ET_c_* and lower *R_e_*—and is further complicated by regional disparities in irrigation infrastructure and groundwater accessibility. In areas with historically abundant groundwater and well-developed irrigation systems, farmers tend to increase irrigation frequency to stabilize yields under rising evaporative demand, leading to sharp increases in *I_wr_*. In contrast, in water-scarce regions where groundwater levels have already declined or strict pumping restrictions are enforced, farmers often reduce irrigation intensity, resulting in a decline in *I_wr_* but also potential yield losses (Zhai et al., 2005). This spatial differentiation in irrigation behavior reveals that groundwater overexploitation is not solely a hydrological problem but is deeply rooted in socio-economic disparities and uneven adaptation capacity. Consequently, one-size-fits-all macro-level policies may prove insufficient to address region-specific irrigation challenges (Liu et al., 2018a, 2018).

### 4.4 Impact of different cropping patterns on groundwater consumption

This study quantitatively confirms winter wheat as the dominant contributor to groundwater depletion in Henan’s double-cropping system, with an *ANGWC* three to four times greater than that of summer maize (Liu et al., 2017). Seasonal fallowing of winter wheat in high-risk zones thus represents a direct and effective measure to reduce abstraction and facilitate aquifer recovery (Bian et al., 2024).

However, the sharp post-2010 increase in summer maize *NGWC* signals a critical shift. This transformation is driven by: (1) Policy-driven expansion and adoption of high-yielding, water-intensive cultivars under grain security initiatives (Yang et al., 2013); (2) Irrigation management shifts, including increased frequency and practices like no-till direct seeding that can disrupt water demand-rainfall synchrony (Scanlon et al., 2012); and (3) Climatic factors like more frequent late-summer droughts. Consequently, winter wheat fallowing policies implemented without parallel regulation of summer maize irrigation may see groundwater savings offset by increased maize water use (Li et al., 2024a).

To ensure sustainable groundwater management, a regionally differentiated dual strategy is proposed. This entails targeted winter wheat fallowing coupled with strict irrigation quotas and water-saving technologies for summer maize in the critically overexploited central-northern plains, while optimizing irrigation scheduling in the wetter south. Effective policy frameworks must integrate fallow subsidies with quota-based water allocation (Pereira et al., 2002; Cao et al., 2020) and promote efficient irrigation technologies and cultivars (Duan et al., 2018). Addressing socio-economic challenges, such as farm income losses and adoption costs, through tailored incentives is paramount for balancing groundwater sustainability with food security and livelihoods (Gleick, 2003).

### 4.5 Research limitations

This study retains certain limitations that point to future research directions. The spatial interpolation using the Inverse Distance Weighting (*IDW*) method, while effective for capturing spatial patterns, may introduce uncertainty in sparsely sampled western mountainous and peripheral areas, as it cannot quantify prediction uncertainty like geostatistical methods (e.g., Kriging) (Vicente-Serrano et al., 2003). Furthermore, the fixed coefficient method for effective precipitation estimation, though operationally simple and suitable for long-term analysis, may introduce systematic biases by not fully accounting for the dynamic impacts of soil hydraulic properties and rainfall intensity on infiltration and runoff (Kim and Stricker, 1996).

## 5 Conclusions

This study investigated the spatiotemporal dynamics of crop water use and groundwater depletion in Henan Province’s winter wheat–summer maize rotation system from 1961 to 2020 using a water balance framework integrating *ET_c_*, *R_e_*, *I_wr_*, and *NGWC*. Results reveal strong temporal and spatial contrasts between the two crops. Winter wheat *ET_c_* increased due to climate warming during spring and early summer, while summer maize *ET_c_* decreased, reflecting improved water-use efficiency and adaptive management. Effective precipitation declined in key growth stages, with rainfall becoming more concentrated and infiltration efficiency reduced, particularly in coarse-textured soils of northern Henan. Consequently, Irrigation water demand decreased for maize but increased for winter wheat, indicating widening water supply–demand disparities. Groundwater depletion is dominated by winter wheat, whose *ANGWC* remains three to four times that of maize. However, the post-2010 increase in summer maize groundwater use signals a shift toward dual-season water stress, especially in the central–northern plains. To ensure sustainable groundwater management, this study recommends a dual strategy integrating winter wheat fallowing and summer maize water conservation. Coordinated policies—combining targeted fallow subsidies, irrigation quotas, and high-efficiency irrigation technologies—are essential for balancing agricultural production with groundwater sustainability. These findings provide a scientific basis for designing adaptive water management strategies in China’s major grain-producing regions.

## Data Availability

The raw data supporting the conclusions of this article will be made available by the authors, without undue reservation.

## Glossary

ET_c_: Crop Evapotranspiration
R_e_: Effective Precipitation
I_wr_: Irrigation Water Requirement
NGWC: Net Groundwater Consumption
ANGWC: Annual Net Groundwater Consumption
ET_0_: Reference Crop Evapotranspiration
FAO: Food and Agriculture Organization of the United Nations
IDW: Inverse Distance Weighting
M-K Test: Mann-Kendall Test
M1: Initial Stage (Summer Maize)
M2: Development Stage (Summer Maize)
M3: Mid-Season Stage (Summer Maize)
M4: Late Season Stage (Summer Maize)
W1: Initial Stage (Winter Wheat)
W2: Development Stage (Winter Wheat)
W3: Mid-Season Stage (Winter Wheat)
W4: Late Season Stage (Winter Wheat)
M1ETc: ETc at the Initial Stage of Summer Maize
W2R_e_: Re at the Development Stage of Winter Wheat
M3I_wr_: Iwr at the Mid-Season Stage of Summer Maize
W4NGWC: NGWC at the Late Season Stage of Winter Wheat
M1981-1990: ANGWC for the period 1981-1990 (Summer Maize)
M1991-2000: ANGWC for the period 1991-2000 (Summer Maize)
M2001-2010: ANGWC for the period 2001-2010 (Summer Maize)
M2011-2020: ANGWC for the period 2011-2020 (Summer Maize)
W1981-1990: ANGWC for the period 1981-1990 (Winter Wheat)
W1991-2000: ANGWC for the period 1991-2000 (Winter Wheat)
W2001-2010: ANGWC for the period 2001-2010 (Winter Wheat)
W2011-2020: ANGWC for the period 2011-2020 (Winter Wheat)
